# Radiation dose, chemotherapy, hormonal treatment and risk of second cancer after breast cancer treatment

**DOI:** 10.1038/sj.bjc.6601138

**Published:** 2003-08-26

**Authors:** C Rubino, F de Vathaire, A Shamsaldin, M Labbe, M G Lê

**Affiliations:** 1 Unité INSERM XUR521, Institut Gustave Roussy, 39 rue Camille Desmoulins, 94805 Villejuif, France; 2 Unit of Radiophysic, Institut Gustave Roussy, Villejuif, France

**Keywords:** breast cancer, second cancer, radiotherapy, dosimetry, chemotherapy, case–control study

## Abstract

In total, 281 of the 7711 women who were initially treated for breast cancer between 1954 and 1983 at the Gustave Roussy Institute developed a second malignant neoplasm (SMN) other than second primary breast cancer and nonmelanoma skin cancer at least 1 year after breast cancer treatment. We carried out a nested case–control study to determine the overall relationship between the dose of radiotherapy received at a given anatomical site and the risk of SMN at the same site. In total, 75% of the cases of SMN were previously treated by radiotherapy, as compared to 73% of the controls. In the irradiated patients, the median local dose was higher among cases (3.1 Gy) than among controls (1.3 Gy). More than 40% of the irradiated patients received a local dose of less than 1 Gy. A purely quadratic relationship was observed between the dose of radiation received at an anatomical site and the risk of SMN at this site. According to the quadratic model, the excess risk of SMN was 0.2% (95% CI 0.05–0.5%) when the target organ received 1 Gy. This risk did not differ significantly according to age at the time of radiotherapy (<40 *vs* ⩾40 years). The risk of SMN was 6.7-fold higher for doses of 25 Gy or more than in the absence of radiotherapy. No carcinogenic effect of chemotherapy was observed and a dose–effect relationship between the length of tamoxifen treatment and SMN occurrence was found. This relationship was limited to endometrial cancers and did not modify the relationship with radiation dose. Our results suggest that high radiation doses slightly increase the risk of second malignancies after breast cancer.

Population- and hospital-based studies suggested that among breast cancer survivors, the risk of developing a second cancer at other sites is 10–40% higher than in the general population (Harvey and Brinton, 1985; Rubino *et al*, 2000). Genetic and hormonal factors may play a role in this increased risk, as indeed may radiation, chemo- and hormonal treatments of breast cancer.

The risk of cancer following ionising radiation has been extensively studied and is relatively well known compared to the risks due to other carcinogens (UNSCEAR, 2000). However, little data are available for breast cancer patients even though radiation therapy is widely used to reduce the risk of local recurrence. The relative risk of second malignant neoplasm (SMN) associated with external radiotherapy is between 0.7 and 1.8 (Harvey and Brinton, 1985; Herring *et al*, 1986; Murakami *et al*, 1987; Lavey *et al*, 1990; Andersson *et al*, 1991; Cuzick *et al*, 1994; Fisher and Anderson, 1994; Valagussa *et al*, 1994; EBCTCG, 2000; Tanaka *et al*, 2001). The conditions in radiotherapy units delivering high doses to limited volumes are quite different from those in the cohorts studied to estimate the risk of ionising radiation (UNSCEAR, 2000). Consequently, predicting the risks of radiation for breast cancer requires an estimate of the relationship between the radiation dose at a given site and the risk of SMN at this site. Owing to the heterogeneity of the distribution of the radiation dose through the body, the overall role of radiotherapy in SMN risk can only be directly investigated by studying this relationship for all the sites of SMN together.

To this end, we performed a case–control study nested in a cohort of 7711 women treated for breast cancer between 1954 and 1983. The general characteristics of this cohort, as well as results for secondary lung cancer have been reported (Rubino *et al*, 2000, 2002). This is the first report concerning the dose of radiation received for breast cancer treatment and the risk of overall second cancer.

## PATIENTS AND METHODS

### Patients

Cases were defined as women who developed an SMN at least 1 year after the diagnosis of breast cancer, among a cohort of 7711 women treated for breast cancer at the IGR between 1954 and 1983. We included all women with histologically confirmed SMN, except those with contralateral breast cancer, nonmelanoma skin cancer and second cancers of unknown origin. The 281 patients who met these criteria were included in the case–control study (Table 1

**Table 1:** Number of cases of each type of SMN, mean age at breast cancer treatment and mean latency between breast cancer treatment and SMN occurrence

**Cancer site**	**ICD-9**	**Number of Cases**	**Mean age at breast cancer treatment (min–max)**	**Mean latency in years (min–max)**
Oral cavity and esophagus and larynx	140–150, 161	11	55 (40–70)	9 (2–25)
Stomach	151	15	58 (44–79)	9 (2–23)
Colon	153	33	57 (37–81)	10 (1–26)
Rectum	154	17	51 (32–75)	11 (1–26)
Liver+gall bladder	155–156	2	57 (49–65)	8 (1–14)
Pancreas	157	5	71 (58–80)	7 (1–16)
Lung and bronchus	162–163	11	50 (3–79)	15 (4–23)
Endometrium uterus	182	42	53 (34–76)	11 (1–33)
Cervix uterus	180	27	49 (16–323)	8 (2–6)
Vulva and vagina	184	3	68 (66–69)	12 (9–16)
Ovaries	183	28	50 (35–69)	11 (1–28)
Bladder	188	6	62 (38–80)	9 (3–16)
Kidney	189	10	55 (37–73)	10 (2–29)
Melanoma	172	14	53 (30–79)	13 (2–32)
Nervous system	191–192	1	40	13
Thyroid	193	8	48 (37–68)	9 (3–27)
Bone and soft tissue	170–171	14	55 (41–77)	11 (3–31)
Myeloma	203	5	58 (49–64)	13 (9–17)
Lymphoma	200–202	15	63 (44–87)	11 (1–30)
Leukaemia	204–208	14	56 (41–71)	11 (1–22)

). Each case was matched with two or three controls from the cohort. Patients were matched for age at first cancer (±6 years) and period of treatment (1954–1963, 1964–1973, 1974–1983). Controls had to be followed up over a period that was at least as long as the interval between the breast cancer and SMN diagnosis in the corresponding case (reference date).

Information concerning the initial characteristics of the breast cancer and the radiotherapy, chemotherapy and hormonal therapy received between the breast cancer diagnosis and the reference date was collected from technical and medical records for each case and control. We thereafter considered the treatment received from the diagnosis of breast cancer until 1 year before the reference date (i.e. the ‘useful period’).

#### Radiation dosimetry

The individual dose was calculated using Dos_EG, a software package that was developed at the IGR for retrospective studies and described in detail elsewhere (Grimaud *et al*, 1994; Diallo *et al*, 1996; Shamsaldin *et al*, 1997, 1998, 2000). The doses absorbed at 151 anatomical sites during external beam radiotherapy were estimated for every patient in the case–control study. The corresponding treatment conditions, generator and energy were considered, including the use of a shielding block, wedge modifications, field shapes and field sizes.

To determine whether a dose–response relationship exists, we determined local doses of radiation, defined as the cumulative dose absorbed at the site of (or closest to the site of) the SMN for a case, and at the same site for the matched controls during the useful period. The location of each SMN was determined from medical records. According to this location, one or more of the 151 points estimates were used to estimate the local dose of irradiation received by each case and their matched controls.

Dose reconstruction was not possible for 11 (5%) cases and 18 (4%) controls treated by external radiotherapy due to the absence of technical data. For one control, who was treated by radiotherapy for castration, the local dose of radiation was considered to be the mean dose received at this site after a castration by radiotherapy in the control group. For the other patients, regression analysis was used to explore a correlation among the cases and the controls between the dose received at the site of SMN (or equivalent site for the controls) and both clinical characteristics and treatment features. For 10 cases and 11 controls, a good fit was obtained (*R*^2^>0.4) and the local dose was calculated from the estimated coefficients for each anatomic site among all the cases and the controls, respectively. For one case and six controls, the mean dose received at the anatomic site among, respectively, all the cases and all the controls was attributed.

#### Chemotherapy

Any chemotherapy for initial breast cancer or its recurrence or distant metastasis prior to the occurrence of the secondary cancer was recorded for the 281 cases, as was any chemotherapy received before the reference date for the controls. The details collected for each course or cycle of chemotherapy included the name and total dose of each drug used. The doses of each drug received by each case or control either as initial treatment or for recurrences of the breast cancer (local relapses or distant metastasis) during the useful period were summed per cycle. Drugs were then classified into four categories according to their known mechanism of action in cells, rather than according to their chemical structure: electrophilic agents, spindle inhibitors, inhibitors of nucleotide synthesis and topoisomerase II inhibitors. To determine the total amount of drug in each treatment category, we converted the dose of each chemotherapy agent from milligrams into moles. This was carried out because one molecule of a given drug generally has one active site, whatever its weight. Even if a particular drug has more than one active site per molecule, the error introduced by this hypothesis is probably lower than that introduced when summing the weights.

#### Hormonal therapy

Data concerning castration (by surgery or radiotherapy), tamoxifen treatment and other treatments such as progestational agents, oestrogen, androgen or corticoids were collected. For each category, the total duration of treatment during the useful period was calculated.

### Methods

Cases and controls were compared using conditional logistic regression methods (Breslow and Day, 1980). Generalised risk models were used to evaluate the shape of the dose–response relationship for radiation dose (Preston *et al*, 1991): linear and quadratic increases in the risk of SMN with the radiation dose were tested and the presence of a negative exponential term to take into account a possible cell killing effect at very high dose was researched. In these models, the odds ratio (OR) is expressed as follows:


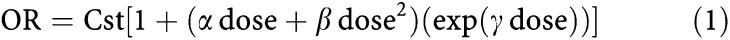


In order to compare our results with those obtained with a single exposure, we used models that took into account the dose per faction and the number of fractions. In these models, the OR associated with n fractions delivering a given dose per fraction dose_f_ is expressed as follows:


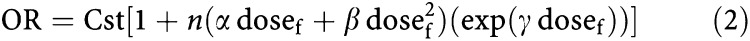


An equivalent formulation of the OR is described in two models (3) and (4) where the OR is expressed as a function of the total dose and the number of fractions,


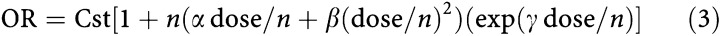



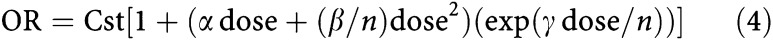


It is noteworthy that these models show that the fractionation effect, if it exists, concerns the quadratic and the exponential terms, but not the linear model. The significance of parameters was tested by comparing nested models. Confidence intervals of the parameters were estimated by likelihood calculations. The analysis was performed using the Epicure epidemiological software (Preston *et al*, 1991).

## RESULTS

### Characteristics of the cases and controls

A significant global heterogeneity was evidenced between the cases according to the type of SMN (Table 1) for age at breast cancer diagnosis (*P*=0.01) and for age at SMN (*P*=0.01), but not for the delay between breast cancer and SMN (*P*=0.2). SMN of the thyroid and cervix uterus occurred at an earlier age than the other types of SMN, among patients who had their breast cancer earlier (Table 1).

Cases and controls were on average 54 years old at the time of breast cancer treatment (ranging from 28 to 87 years), which occurred on average in 1976 (1954–1983). The SMN occurred on average 10 years after breast cancer treatment (up to 33 years). Clinical characteristics of cases and controls were very similar: 76% of cases and 78% of controls had a stage ⩽2 (i.e. M0/N0/T0 to T3, or M0/N1/T0 to T2) cancer, by the UICC classification; 12 cases and 21 controls were M1 (five cases and 10 controls had a primary bilateral breast cancer, and seven cases and 11 controls had metastasis). At the time of breast cancer diagnosis, half of the cases and controls had no ovarian activity.

About three-quarters of the cases and controls underwent mastectomy and less than one-fifth underwent conservative surgery (lumpectomy or partial mastectomy); 209 (74%) of the cases and 443 (72%) of the controls received radiotherapy (Table 2

**Table 2:** Details of the breast cancer treatments received by the patients

	**Cases**	**Controls**
**Treatment characteristics**	**(*N*=281)**	**(*N*=614)**
*Loco-regional treatments*		
Radiotherapy^a^: number of patients(%)	209 (74)	443 (72)
Fractions: mean (range)	27 (2–78)	28 (4–189)
Dose (Gy) to the breast^b^: median (min–max)	52.1 (19.7–112)	54.0 (12.4–117)
Dose (Gy) to the site of SMN^c^: median (min–max)	3.1 (0.01–68.4)	1.3 (0.002–79.8)

*Systemic treatments*		
Chemotherapy^d^: number of patients (%)	29 (10)	62 (10)
Electrophilic agents	26 (9)	58 (9)
Spindle inhibitors	25 (9)	45 (7)
Inhibitors of nucleotide synthesis	29 (10)	60 (10)
Topoisomerase II inhibitors	19 (7)	40 (7)

Hormonal treatment^e^: number of patients (%)	71 (25)	132 (22)
Tamoxifen	46 (16)	93 (15)
Progesterone	13 (5)	35 (6)
Oestrogens	6 (2)	11 (2)
Androgen	25 (9)	27 (4)
Corticoid	5 (2)	10 (2)
Other hormonal treatments	5 (2)	11 (2)

* Castration: number of patients* (%)		
* Radiotherapy*	68 (24)	150 (24)
* Surgery*	8 (3)	24 (4)

a Including treatments for distant metastases and castration; 48 cases and 105 controls were treated with several machines and the type of machine was unknown for 11 cases and 18 controls.

b Cumulative dose of radiation received to the site of the breast treated for a breast cancer.

c Cumulative dose of radiation received to the same site of the breast for the matched controls.

d Each patient received one or more types of treatment.

). Radiotherapy was performed with Cobalt-60 gamma rays in 96% of the cases and 97% of the controls, associated with electron beams in 24% of the cases and 22% of the controls. Only 3% of the cases and 5% of the controls were treated with low-energy X-rays produced by orthovoltage machines (200–250 KV) and 3% of the cases and 2% of the controls with megavoltage X-rays (4–20 MeV). Although the cumulative dose to the breast and the number of fractions were very similar in cases and in controls, the local dose was about three times higher among cases (3.1 Gy in median) than controls (1.3 Gy).

In total, 10% of the cases and controls were treated by chemotherapy. The treatment protocols associated mostly two or more of the following drugs: cyclophosphamine, 5 fluorouracil, methotrexate, adriamycin and vincristine. Each group of chemotherapy drug was given to an approximately equal proportion of cases and controls, with no major differences in the mean number of moles administered between the two groups.

Hormonal treatment with one or more drugs was prescribed to 25% of the cases and 22% of the controls. About 15% of the cases and controls received tamoxifen, with a treatment duration about two times higher among cases (43 months in median) than controls (27 months). This hormone was either administered for the initial treatment of advanced cancer among postmenopausal or for the treatment of relapses. The other hormones administered were mostly progesterone and androgens. Progesterone was mainly prescribed to women around 50 years old, who were not receiving any other hormone treatments, whereas androgens were mostly given to older women in association with tamoxifen for the treatment of distant metastases.

Ovarian function was suppressed in 76 cases and 174 controls, by pelvic radiotherapy in 68 cases (24%) and 150 (24%) controls. The remaining eight cases and 24 controls underwent surgery.

### Risk associated with radiotherapy

The overall OR of SMN associated with initial radiotherapy was 1.1 (95% CI: 0.8–1.6). A significant dose–response relationship was found between the radiation dose to the anatomic site of SMN and the risk of SMN (*P*<0.01), as shown in Table 3

**Table 3:** Odds ratios (OR) of second cancer as a function of the local dose of radiation

**Local radiation dose in Gy**	**Number of cases/controls**	**Mean dose in controls**	**Unadjusted OR (95% CI^b^)**	**Adjusted OR^a^ (95% CI^b^)**	**Test of trend^c^ *P*-value**
0	72/171	0	1 (ref)	1 (ref)^c^	
]0–1[	94/212	0.19	1.1 (0.8–1.7)	1.1 (0.7–1.6)	
[1–5[	20/56	2.7	0.9 (0.4–1.6)	0.8 (0.4–1.6)	
[5–10[	30/54	7.3	1.3 (0.7–2.5)	1.2 (0.6–2.2)	<0.01
[10–15[	30/69	12.9	1.1 (0.6–1.9)	1.1 (0.6–1.9)	
[15–20[	16/34	15.9	1.3 (0.6–2.8)	1.3 (0.6–2.8)	
[20–25[	4/3	22.9	4.5 (0.9–27.2)	4.0 (0.8–23.2)	
⩾25	15/15	41.1	6.4 (1.8–31.4)	6.7 (1.9–33.2)	
Any dose	209/443	6.1	1.14 (0.82–1.59)	1.11 (0.80–1.56)	

a OR adjusted for duration of tamoxifen treatment in months and chemotherapy (Y/N).

b 95% CI=95% confidence interval.

c Test of trend using a quadratic dose–effect relation, after adjustment for duration of tamoxifen treatment in months and chemotherapy (Y/N).

. Different dose–responses relationships were considered (Table 4

**Table 4:** OR models and regression coefficients of second cancer for the local dose of radiation in Gy, adjusted for chemotherapy administration (yes/no) and for duration of tamoxifen treatment

	**Regression coefficients**			
**Models**	***β*_1_**	***β*_2_**	***γ*_1_**	**Deviance**
Baseline: OR=exp(*α*_1_ chemo+*α*_2_ tmx)	—	—	—	642.2
Linear: OR=exp(*α*_1_ chemo+*α*_2_ tmx) × [1+*β*_1_(dose)]	0.04	—	—	634.4
Quadratic: OR=exp(*α*_1_ chemo+*α*_2_ tmx) × [1+*β*_2_(dose × dose)]	—	0.002	—	631.0
Linear-quadratic: OR=exp(*α*_1_ chemo+*α*_2_ tmx) × [1+*β*_1_(dose)+*β*_2_(dose × dose)]	−0.03	0.003	—	630.6
Linear-quadratic-exponential: OR=exp (*α*_1_ chemo+*α*_2_tmx) × [1+((*β*_1_(dose)+*β*_2_ (dose × dose)) × exp (*γ*_1_ × dose)]	−0.02	0.003	0.01	630.6

**Figure 1:**
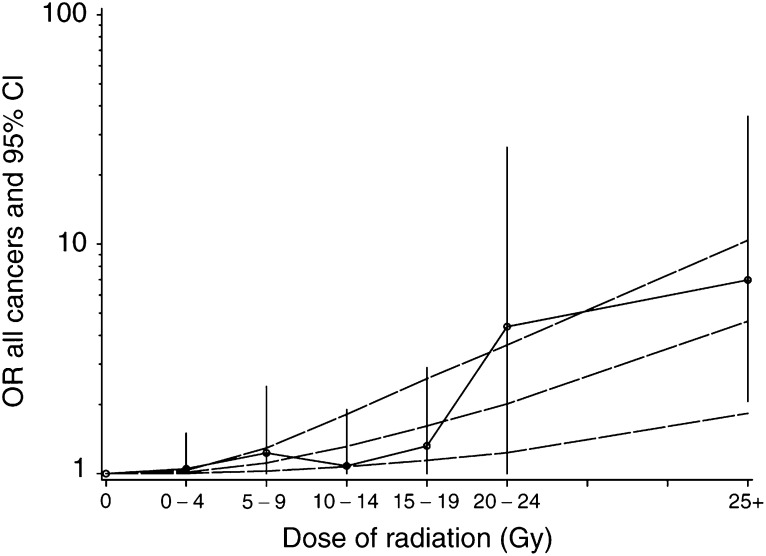
OR of second malignant neoplasm (SMN) as a function of the radiation dose received to the site of the SMN for cases and the equivalent site for controls (with 95% CI). The curves correspond to the estimated excess of the OR of SMN as a quadratic function of the radiation dose (dotted curves: upper and lower 95% CI).

Chemo=chemotherapy; tmx=tamoxifen.

). The best fit was obtained for a purely quadratic dose–response relationship, without a negative exponential term for cell killing at high doses. Indeed, the deviance of the quadratic model (deviance=631.0) was lower than that of the linear model and was not significantly reduced by the addition of a linear term (deviance=630.6) or both linear and exponential terms (deviance=630.6). The estimated excess odds ratio for a dose of 1 Gy was 0.002 (95% CI: 0.0005–0.005) with the quadratic model. Figure 1

illustrates the dose–effect relationship with the expected values according to a quadratic dose–effect model. These results were verified taking the effects of latency, age at diagnosis of breast cancer, type of SMNs and effect of fractionation into account successively.

The results were similar after excluding the 85 cases of SMN that occurred during the first 5 years after breast cancer treatment and their matched controls: the best fit was obtained with a purely quadratic model and the OR at 1 Gy estimated with a quadratic model was similar to the previous estimate: 0.003 (95% CI: −0.0006 to 0.007).

Only 26 cases were less than 40 years old at the time of breast cancer treatment. Among these women and their 64 controls, the overall OR associated with initial radiotherapy was 2.5 (95% CI: 0.7–11.4), not significantly different from that in older patients (*P*=0.2). In the same way, the dose–response for radiation dose was not significantly different in these two groups (*P*=0.3).

Our case–control study included 11 patients with lung cancer and 14 with soft tissue and bone cancer. A dose–response relationship has previously been described for each of these two types of SMN. After excluding these cancers, the OR for radiotherapy was 1.0 (95% CI: 0.7–1.5), without a significant dose effect–relationship (*P*=0.3), and the OR at 1 Gy estimated with a quadratic model was 0.0006 (95% CI: −0.001 to 0.003). No significant, or near significant, dose–response was evidenced for any of the other sites of SMN.

A purely quadratic model fitted the OR as a function of the dose per fraction (model (2)). This result was not modified when a linear (*P*=0.7) and/or an exponential term (*P*=0.6) were added. The excess of odds ratio at 1 Gy per fraction was 0.034 (95% CI: 0.007–0.09) with a quadratic model.

### Effect of chemotherapy

No carcinogenic effect of chemotherapy was evidenced: the OR associated with chemotherapy was 0.8 (95% CI: 0.5–1.2) and none of the four drug categories were significantly associated with the occurrence of SMN, even after adjustment for radiotherapy and hormonal therapy.

### Effect of hormonal therapy

The overall OR of SMN associated with tamoxifen treatment was 1.2 (95% CI: 0.7–1.9). A significant dose–response relationship was found between the cumulative length of treatment and the risk of SMN occurrence (*P*=0.03) as shown in Table 5

**Table 5:** OR of SMN as a function of the total duration of tamoxifen treatment, adjusted for radiation dose and chemotherapy administration (yes/no)

	**All SMNs**		**All SMNs excluding endometrial cancer (*N*=239)**		**Endometrial cancer (*N*=42)**	
**Duration of tamoxifen treatment in months**	**Cases/controls**	**OR (95% CI)**	**Cases/controls**	**OR (95% CI)**	**Cases/controls**	**OR (95% CI)**
None or less than 1	236/522	1 (ref)*	204/426	1 (ref)**	32/96	1 (ref)***
[1–24[	20/52	0.9 (0.5–1.7)	19/39	1.0 (0.5–1.9)	1/13	0.3 (0.01–1.4)
[24–48[	9/22	0.9 (0.4–2.1)	7/19	0.7 (0.3–1.7)	2/3	1.4 (0.2–9.0)
[48–72[	8/15	1.2 (0.4–3.1)	9/15	1.2 (0.5–2.9)	7/3	13.1 (2.2–249)
⩾72	8/3	5.9 (1.6–28.2)}				

* Test of trend=*P*<10^−2^;

** test of trend=*P*=0.03;

*** test of trend=*P*=0.4.

. However, this association was restricted to endometrial cancers, that is, 42 of the 281 cases. In this group, the risk of SMN was 21.3-fold higher (95% CI: 2.4–563) after 4 years of treatment compared to in the absence of such treatment. Among the cases with second malignancies at other sites, the duration of tamoxifen treatment had no effect (Table 5).

### Interactions between radiotherapy and systemic treatments

No significant interaction was observed between radiation dose and chemotherapy or tamoxifen administration. The odds ratio for a local dose of radiation higher than 1 Gy associated with chemotherapy was 1.0 (95% CI: 0.5–1.9), compared to a null or lower radiation dose in the absence of chemotherapy (Table 6

**Table 6:** OR of SMN according to systemic treatment and radiotherapy dose received at the SMN site of the case and at the same site for matched controls

	**Dose of radiotherapy at the SMN site**					
	**0–1 Gy**		**>1 Gy**		**Total**	
**Systemic treatment**	**OR**	**(No.)**	**OR**	**(No.)**	**OR**	**(No.)**
*Chemotherapy*						
No	1a	(152/357)	1.2	(100/195)	1b	(252/552)
Yes	1.3	(14/26)	1.0	(15/36)	1.0	(29/62)

*Tamoxifen*						
No	1a	(140/327)	1.2	(95/194)	1b	(235/521)
Yes	1.1	(26/56)	1.3	(20/37)	1.1	(46/93)

No.=Number of cases of SMN/ number of controls. 1a=Reference category for the risks according to radiotherapy dose and systemic treatment. 1b=Reference category for the risk of systemic treatment, adjusted for radiotherapy dose.

). Similarly, the odds ratio associated with doses over 1 Gy and tamoxifen was 1.3 (95% CI: 0.7–2.3). The estimates of the radiation dose–response relationship were not modified by the adjustment for chemotherapy and/or duration of tamoxifen treatment.

## DISCUSSION

Our case–control study of 281 cases of SMN and 614 controls, nested in a cohort of 7711 women treated for breast cancer at the IGR between 1954 and 1984 showed that radiation increased the risk of SMN. A quadratic relationship was found between the dose of radiation received at a given anatomical site and the risk of SMN occurrence at this site. The radiation-induced risk was largely limited to lung cancer and bone and soft-tissue sarcoma, and the dose–response relationship was no longer significant when these two types of cancer were excluded. No carcinogenic effect of chemotherapy was observed and there was a dose–effect relationship between the length of tamoxifen treatment and SMN occurrence. This relationship was limited to endometrial cancers and did not modify the relationship with radiation dose. This study is the first report on the relationship between the dose of radiation received for breast cancer treatment and the risk of overall second cancer.

The dose–response relationship observed has to be interpreted carefully. We estimated the average relationship between the radiation dose at a set of anatomical sites (or organs, if small and equivalent to a point) and the risk of SMN at the same sites. In the case of whole body homogeneous irradiation, this dose–response relation would directly predict the overall excess cancer risk for such a dose. In the case of radiotherapy, this dose–response relationship is still an accurate estimate of the average dose–response for all cancer sites together, but it cannot be used to predict the overall risk of radiation-induced cancer in patients.

We estimated that the excess risk of SMN when 1 Gy was delivered to the target organ was 0.2% (95% CI: 0.05–0.5%) when the total dose was delivered in an average of 28 fractions. This is far from that estimated from risk coefficients established with Hiroshima and Nagasaki (HN) survivors, even when the age of the women in our cohort at the time of breast cancer treatment was taken into account. For HN survivors, there is a linear relationship between dose of radiation and risk of solid tumours and the excess relative risk per Gy among women aged 30 years at the time of irradiation is 79% for all solid cancers (UNSCEAR, 2000). The excess relative risk decreases by 50% for each additional decade of age at the time of irradiation and is thus about 10% at 55 years of age. The risk we estimated for the total dose of radiation is an estimation of the risk associated to the entire radiation treatment in several fractions and is not directly comparable to the risk induced by a single exposure to radiation. Conversely, the excess risk we estimated at 1 Gy per fraction, that is, 3.4% (95% CI: 0.7–9%), which can be compared to that of a single exposure, is lower but probably not statistically different from the estimation for HN survivors (10%). In addition, due to the quadratic shape of the relationship, this discrepancy reduces with increasing dose. Thus, for example, for a dose per fraction of 2 Gy, the excess OR we estimated, that is, 14% (95% CI: 2.8–36%), is not significantly different from that estimated from HN-survivors with a linear model (20%).

In general, excess risks per dose unit in studies of cancer risk following radiotherapy are lower than those estimated in HN survivors (Little, 2001). Little investigated this finding in detail and concluded that this discrepancy can largely be explained by cell sterilisation effects, although other factors such as difference in underlying cancer risk and dose fractionation effects may also contribute (Little, 2001). Conversely, our results do not support a role of cell sterilisation. This was nonsignificant, whatever the model fitted. Instead, they support the hypothesis that this discrepancy is due to dose fractionation.

The risk associated with radiotherapy in our case–control study is lower than that previously estimated in a sub-cohort analysis including 4416 women (Rubino *et al*, 2000): 1.1 *vs* 1.6. This discrepancy is not due to overmatching, but is explained by the patient selection in the sub-cohort. The low OR we estimated for radiotherapy (yes/no), 1.1, is consistent with the low estimated coefficient for dose–response, and with the general findings of 11 other studies on SMN incidence or mortality (0.7–1.8). Only three studies (Herring *et al*, 1986; Lavey *et al*, 1990; Fisher and Anderson, 1994), the smallest ones, which included less than 50 SMN cases, estimated a relative risk of above 1.5. The other studies (Harvey and Brinton, 1985; Murakami *et al*, 1987; Andersson *et al*, 1991; Cuzick *et al*, 1994; Valagussa *et al*, 1994; EBCTCG, 2000; Tanaka *et al*, 2001; Veronesi *et al*, 2002) included about 3500 SMN incident cases or deaths and estimated a maximum relative risk of 1.2. The RR associated with radiotherapy generally estimated in previous studies is probably very similar to our finding.

We did not find that the risk of SMN was increased by chemotherapy administration. Chemotherapy was first used as part of the treatment of breast cancer during the late 1970s. Consequently, only a small proportion of the women in our study (10%) received this treatment. At present, eight studies have investigated the role of chemotherapy in the risk of all types of SMN. In two studies (Lavey *et al*, 1990; Rubagotti *et al*, 1996), which included a total of 59 SMN cases that occurred during an average follow-up of 5 years, the relative risk associated with chemotherapy was between 2 and 3. In the other six studies (Herring *et al*, 1986; Murakami *et al*, 1987; Valagussa *et al*, 1987, 1994; Matsuyama *et al*, 2000; Tanaka *et al*, 2001), this risk ranged from 0.5 to 1.05. Overall, there is currently no evidence that the overall SMN risk is increased by the antineoplasic drugs administered for breast cancer treatment. However, new drugs are regularly introduced and no published study has a long enough follow-up period to study the risk associated with recent drugs.

Tamoxifen is increasingly used as adjuvant therapy and reduces recurrences and mortality among breast cancer patients as well as the occurrence of contralateral breast cancers (EBCTCG, 1998). We found a low increased risk associated with this hormonal therapy, limited to endometrial cancer, as previously shown (Curtis *et al*, 1996; EBCTCG, 1998; Mignotte *et al*, 1998). Tamoxifen treatment increases the risk of overall SMN by 0.7–1.2 depending on the study (Fornander *et al*, 1989; Andersson *et al*, 1991, 1992; Rutqvist *et al*, 1995; Curtis *et al*, 1996; Rubagotti *et al*, 1996; EBCTCG, 1998; Newcomb *et al*, 1999; Tanaka *et al*, 2001).

In conclusion, our results suggest that radiotherapy plays a small role in the overall risk of second malignancies after breast cancer.
